# PE_PGRS proteins of *Mycobacterium tuberculosis*: A specialized molecular task force at the forefront of host–pathogen interaction

**DOI:** 10.1080/21505594.2020.1785815

**Published:** 2020-07-25

**Authors:** Flavio De Maio, Rita Berisio, Riccardo Manganelli, Giovanni Delogu

**Affiliations:** a Dipartimento di Scienze di Laboratorio e Infettivologiche, Fondazione Policlinico Universitario “A. Gemelli”, Rome, Italy; b Dipartimento di Scienze Biotecnologiche di Base, Cliniche Intensivologiche e Perioperatorie – Sezione di Microbiologia, Università Cattolica del Sacro Cuore, Rome, Italy; c Institute of Bio-Structures and Bio-Imaging - CNR-IBB, Naples, Italy; d Department of Molecular Medicine, University of Padova, Padua, Italy; e Mater Olbia Hospital, Olbia, Italy

**Keywords:** PE/PPE proteins, PE_PGRSs, *Mycobacterium tuberculosis*, mycobacterial envelope, bacterial pathogenesis

## Abstract

To the PE_PGRS protein subfamily belongs a group of surface-exposed mycobacterial antigens that in *Mycobacterium tuberculosis* (*Mtb*) H37Rv accounts to more than 65 genes, 51 of which are thought to express a functional protein. PE_PGRS proteins share a conserved structural architecture with three main domains: the N-terminal PE domain; the PGRS domain, that can vary in sequence and size and is characterized by the presence of multiple GGA-GGX amino acid repeats; the highly conserved sequence containing the GRPLI motif that links the PE and PGRS domains; the unique C-terminus end that can vary in size from few to up to ≈ 300 amino acids. *pe_pgrs* genes emerged in slow-growing mycobacteria and expanded and diversified in MTBC and few other pathogenic mycobacteria. Interestingly, despite sequence homology and apparent redundancy, PE_PGRS proteins seem to have evolved a peculiar function. In this review, we summarize the actual knowledge on this elusive protein family in terms of evolution, structure, and function, focusing on the role of PE_PGRS in TB pathogenesis. We provide an original hypothesis on the role of the PE domain and propose a structural model for the polymorphic PGRS domain that might explain how so similar proteins can have different physiological functions.

## Introduction

*Mycobacterium tuberculosis* (*Mtb*) is one of the ancients and most successful human pathogens, still responsible for ≈ 10 million active TB cases and ≈ 1.5 million deaths in 2018 [1]. The most common outcome following *Mtb* infection is latent TB (≈ 95%), with no signs or symptoms of disease, characterized by a dynamic equilibrium between the host immune response and the bacillus which usually last for lifetime [2–4]. The immunological mechanisms governing the host-*Mtb* interaction remain poorly understood as well as the bacterial proteins and virulence factors that provide *Mtb* with these unique features [5,6]. *Mtb* belongs to the species *Mycobacterium tuberculosis* complex (MTBC), which is a monomorphic species subdivided in phylogeographic lineages that also include: *M. africanum*, that causes TB in humans but only in certain regions of Africa; *M. microti*, that causes TB in voles; *M. bovis*, that comprises several ecotypes that cause TB in wild and domesticated animals [7]. Comparative genomics between *Mtb*, or rather MTBC, and other mycobacterial species (most importantly with *Mtb* progenitors as the smooth tubercle bacilli), indicated that the evolution of *Mtb* as a human pathogen has been mainly characterized by a process of gene loss and intragenomic recombination [8–10].

Interestingly, among the gene families that are restricted and abundant in *Mtb* are the *pe* and *ppe* genes, which evolved through multiple events of gene duplication, recombination and diversification, and occupy almost 10% of the *Mtb* genome coding capacity [11–13]. PE proteins are divided in three subfamilies: PE-only, which are usually less than 100 amino acids in length and are associated with a PPE protein; PE_unique, which present downstream of the PE domain a unique amino acid sequence of variable sequence; and PE_PGRS which contain the polymorphic glycine-rich domain of variable sequence and size. Of interest, a remarkable feature of two protein subfamilies, PE_PGRS, and PPE_MPTR, is the presence of repeated and apparently redundant sequences at the protein C-terminus, with very little or no homology with other proteins [14].

The polymorphic GC-rich sequences (PGRS), which were first identified as repetitive genetic sequences and used as a typing tool in molecular epidemiology studies [15], were discovered as proteins-coding sequences only following completion and assembly of the *Mtb* genome [11]. The *Mtb* genome contains 65 *pe_pgrs* genes, although only 51 of these express a functional protein, at least in *Mtb* H37Rv [16]. These genes are found in all members of MTBC and few other mycobacterial species that can cause diseases in humans as *M. marinum* (≈ 148 genes) and *M. ulcerans* (≈ 121 genes), although *pe_pgrs* genes in these species show significant differences with those found in MTBC [14,17]. It is widely accepted that PE_PGRS proteins are important in TB pathogenesis, yet their functional and biological role remains elusive [18,19]. Here, we summarize the current knowledge on these proteins and provide a hypothesis on their role in TB pathogenesis.

## Evolution

**Figure 1.**
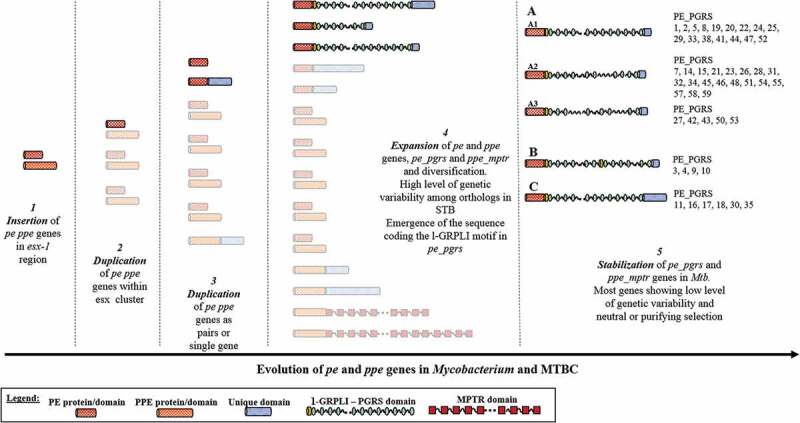
Schematic representation of the evolution of *pe/ppe* genes in the *Mycobacterium tuberculosis* complex (MTBC).

Reconstruction of the genetic relationship of *pe* and *ppe* genes within the mycobacterial genus led to the identification of five coevolving gene subfamilies [14], with the fast-growing mycobacterial species as *Mycobacterium smegmatis* and *Mycobacterium abscessus*, the closest to the genus common ancestor, possessing only few of these genes and some slow-growers species bearing tens of these genes [14]. The progenitors of the *pe* and *ppe* families appeared first associated with the *esx*-1 genetic locus coding the prototype of Type 7 Secretion Systems (T7SS) [14,20]. Duplication of the *esx*-1 cluster followed by multiple duplication events of the *pe* and *ppe* genes led to the expansion of these families in the slow growing mycobacterial species (Figure 1). Pathogenic species as *M. marinum, M. ulcerans,* and MTBC possess a high number of *pe* and *ppe* genes, with an abundance of the most polymorphic *pe_pgrs* and *ppe_mptr* genes belonging to the subfamily V [14]. Interestingly, the appearance and expansion of *pe_pgrs* and *ppe_mptr* genes followed the emergence of *esx*-5 genetic locus, further supporting the close genetic and functional relationship between ESX-T7SS and PE/PPE proteins [14,21,22].

*pe/ppe* genes first emerged in the mycobacterial genome in the *esx-1* locus and evolved following a series of duplication events in the homologous *esx* loci and then spread in the genome as single genes or paired. The combination of duplication and intragenomic recombination events led to the amplification of *pe* and *ppe* genes and the emergence of the *ppe_mptr* and *pe_pgrs* genes, characterized by the presence of repetitive motifs. Based on the features of the PGRS domain and the downstream C-terminal domain, PE_PGRS proteins can be further subdivided in three group (A, B, and C) [16].

Comparative genomic studies in MTBC and in smooth tubercle bacilli (STB) highlighted several examples of gene duplication events, including for instance the *pe_pgrs*17/-18 [23], *pe_pgrs*3/-4, and *pe_pgrs*50/-51 clusters. Intragenic and intergenic recombination in *pe_pgrs* genes was associated with mutations and indels that led to the expansion and diversification of *pe_pgrs* genes. However, it is not clear how horizontal gene transfer processes might have contributed to the expansion and diversification of *pe_pgrs* genes in STB [10]. The genome of recently identified mycobacterial species that are genetically closer to MTBC than *M. marinum*, as *M. riyadhense, M. lacus, M. decipiens,* and *M. shinjukuense* highlighted the presence of at least some *pe_pgrs* genes [24,25]. These newly identified mycobacterial species constitute a common clade with MTBC (MTB-associated phylotype) and it has been proposed that the ancestral founders of this lineage acquired specific genetic features that shaped host-adaptation and virulence [24]. A fine characterization of *pe_pgrs* genes in these mycobacterial species belonging to the MTB-associated phylotype will shed new light on the role of these genes in the evolution of MTBC.

The monomorphic genetic features of MTBC may have supported the stabilization of the *pe_pgrs* genes that continued to evolve at a slower pace by intragenomic rearrangements, mutations, and indels (Figure 1). Interestingly, these genetic events led to the emergence of some genes as for instance *pe*_*pgrs*33, that are unique for MTBC [10]. Homologous recombination of *pe_pgrs* genes shaped the evolution of STB and MTBC, providing the raw material to promote functional innovation and adaptation to the human and more in general mammal host [10,26–29].

Comparison of the genetic sequences of *pe_pgrs* genes among clinical isolates of *Mtb*, or other MTBC strains, indicate that these genes, contrary to what previously proposed, are subject to purifying selection and are highly conserved [30,31]. These findings indicate that there is a strong selective pressure to preserve *pe_pgrs* genes in MTBC, where they must play a key role in the tubercle bacilli biology and TB pathogenesis.

## Structural features

**Figure 2.**
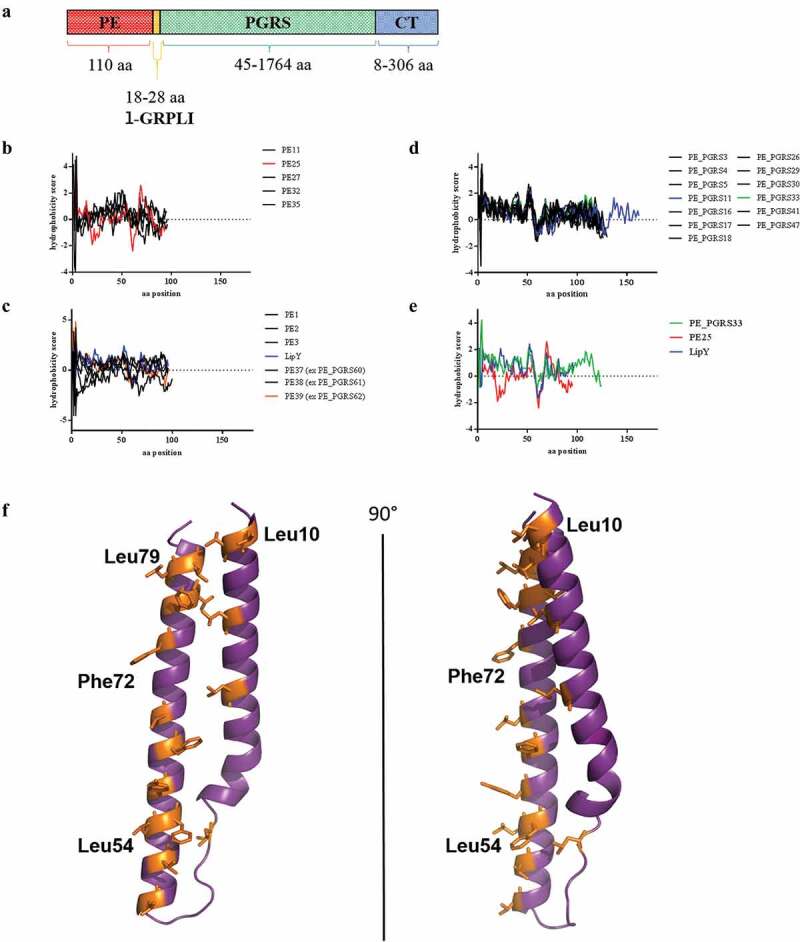
Domain organization of PE_PGRS proteins and hydrophobicity score of PE domains.

PE_PGRS proteins share the same molecular architecture as shown in Figure 2a, characterized by the presence of four main domains: the PE domain, the PGRS, the linker region with the typical GRPLI motif and the unique C-terminal domain.

Schematic showing the typical domain organization of PE_PGRS proteins (A). Hydrophobicity scores, as assessed by *ProtScale Expasy Tool* with *Kyte and Doolitlle* scale, of the PE domain of: PE proteins belonging to the PE/PPE subfamily (B); PE-unique proteins (C); PE_PGRS proteins (D). Panel E shows the hydrophobicity scores for three selected PE proteins belonging to three different subfamilies: PE25 of the PE/PPE pairs; LipY of PE-unique and PE_PGRS33 of the PE_PGRS subfamily. Panel F: cartoon representation of the homology model of the PE domain of PE_PGRS30 in two orthogonal orientations. The model (including residues 8–84) was obtained using MODELER [38]. Hydrophobic and aromatic residues are shown in orange ball-and-stick representation.

### PE domain

The PE domain is ≈ 100 amino acids in length and gives the name to the PE family with the conserved Pro-Glu (PE) amino acids at position 7–8 [11]. The PE-only proteins are ≈ 100 amino acids in length and form a dimer with a PPE protein partner (PE/PPE) [32,33]. The proteins of the PE-unique subfamily have a unique C-terminal domain downstream the classical PE domain that varies in size and function, as, for example, the protein LipY that contains a domain with a lipase activity [34].

Most of the knowledge on the structure and function of the PE domain comes from studies on PE/PPE protein couplets [32,35], which demonstrated that the PE/PPE form dimers that are secreted through the ESX-T7SS [21]. In line with these findings, the ancestral *pe*/*ppe* genes are evolutionary associated with the ESX gene locus and PE/PPE dimers are indeed structurally similar to the ESXA/B and homologous proteins, which are actively secreted antigens known for their immunogenicity [36,37]. The structural similarities and functional association between these ESX substrates have relevant implications in terms of pathogenesis and evolution, as clearly highlighted in dedicated reviews [14,22].

Interestingly, despite a significant degree of amino acids sequence homology, the PE domain of different proteins within the PE/PPE and PE-unique subfamilies show a certain degree of variability, as observed by the poorly conserved hydrophilic/hydrophobic profiles of the amino acidic residues (Figure 2b,c). Conversely, the PE domains of PE_PGRS proteins shows a highly conserved hydrophilic/hydrophobic profile (Figure 2d). The lack of structural studies on the PE domain of PE_PGRS proteins prevents to understand the significance of this observation, yet it is conceivable that it is more structurally constrained than that found in the other PE proteins, although the functional implications remains to be determined (Figure 2e). Sequence identities between PE from PE_PGRS and that from structurally elucidated PE/PPE protein complex (Rv2431/Rv2430) of *Mtb* allow for the determination of homology model structures. An example of homology model computed with MODELER software [38] using the structure of Rv2431 as a template (sequence identity 30%), is reported for PE_PGRS30 in Figure 2f. A clear feature of this structure is the localization of hydrophobic and aromatic residues on one side of the molecule, a typical trait of proteins interacting with other molecules, as in the case of PE/PPE proteins [22,39]. It would be interesting to assess whether the PE domain of PE_PGRS proteins requires a protein partner, similarly to the PPE partner of PE-only protein in PPE41/PE25 dimer [32]. Recent findings suggest that LipY, a PE-unique protein, does not require a partner to be secreted [40]. We suggest a model where PE_PGRSs can be stable on their own, e.g. upon dimerization, which would be in line with the expression of *pe_pgrs* genes as single operons.

### PGRS

The presence of multiple repeats containing the GGA-GGX motif interspersed with unique sequences is the feature of the PGRS domain [12]. The PGRS domain can vary in size from few tens amino acids to almost 1800 (Figure 2a), to form repetitive and apparently redundant mini domains.

**Figure 3.**
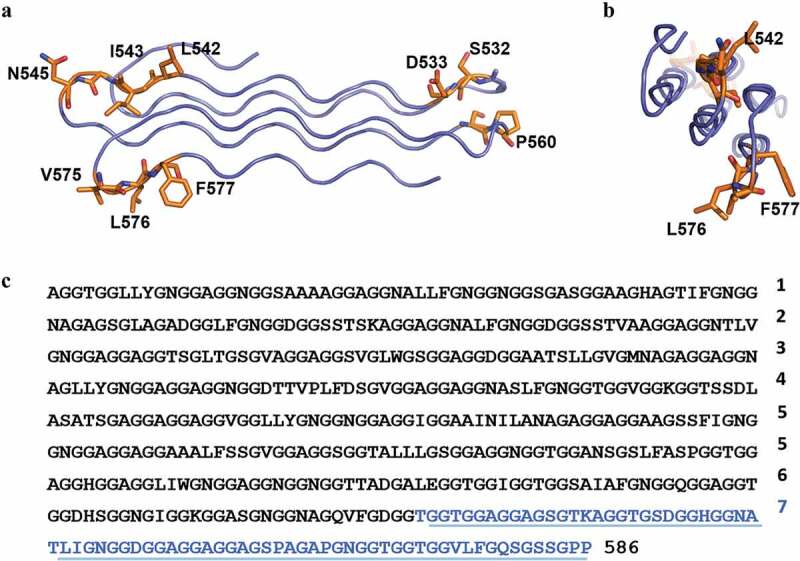
A PG_II_ sandwich domain of PE_PGRS30.

Several studies demonstrated that the PGRS domain is available on the mycobacterial surface and can directly interact with host components, as the TLR2 receptor, implicating these proteins in TB pathogenesis [41–44]. The fact that the PGRS was the target of the host humoral response in TB patients and that PE_PGRS appeared to be polymorphic suggested an involvement of these proteins in the immune evasion strategies [45]. However, more recent evidences indicate that *pe_pgrs* genes are highly conserved and are subjected to purified selection in *Mtb* [30,44], questioning the role of PE_PGRS in antigenic variation. The difficulties in purifying a PE_PGRS protein with a sufficiently large (few hundreds amino acids) PGRS domain have so far hampered their structural and functional characterization, although it is expected that the PGRS is endowed with strong structural flexibility. Indeed, GGAGGX regions are known to induce polyglycine type II-like conformations (PG_II_) [46]. PG_II_, like polyproline type II-like (PP_II_), form flexible left-handed extended helices, which are not constrained by intra-helix hydrogen bonding as in the case of alpha helices. To gather information on the structural features of PGRS domains, homology modeling is extremely useful. Indeed, using the PGRS domain of PE_PGRS30 as a case study, consensus-based sequence alignment using HMMer identifies a structure (PDB code 2PNE, seqid 49% with residues 512–586). Using this alignment, a reliable homology model can be obtained with MODELER [38], that is a compact module composed of five tightly packed PG_II_ helices (Figure 3a,b). As in ideal PG_II_, each helix has three residues per turn and the shape of a triangular prism [47] (Figure 3b). This compact module, denoted as PG_II_ also exists in the Salmonella phage S16 tail fiber adhesin, where the sequence plasticity of the adhesin distal part, involved in the interaction with the bacterial receptor, is ensured by the PG_II_ sandwich [48]. Consistently, PG_II_ like structures have been proposed to mediate protein–protein host–pathogen interactions [49].

Ball-and-stick and cartoon model of a the PG_II_ sandwich domain of PE_PGRS30, embedded between amino acid residues 512 and 586. Panels A and B show two perpendicular views. C) amino acidic sequence of the PGRS domain of PE_PGRS30 and highlighted in blue the sequence corresponding to PG_II_ shown in A and B.

**Figure 4.**
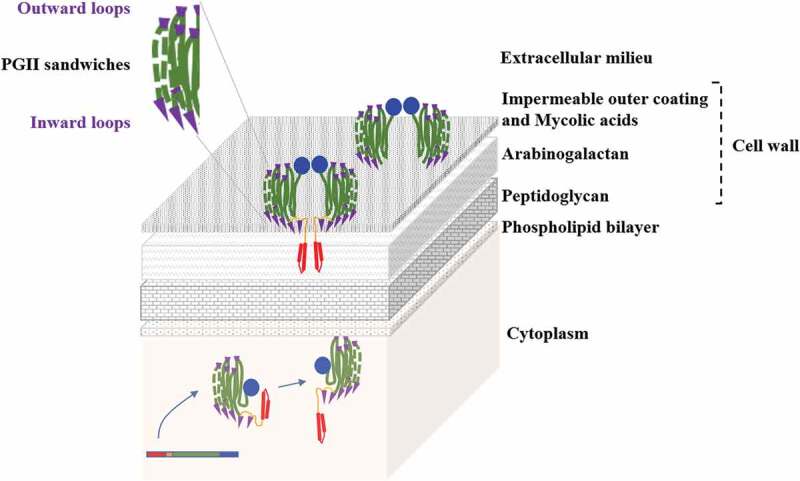
Schematic representation of the PE_PGRS localization in the mycobacterial cell wall.

Interestingly, an analysis of the PE_PGRS sequences suggest that these proteins may contain a variable number of PG_II_ sandwich modules. In the case of PE_PGRS30, we predict the existence of eight PG_II_ sandwich domains, each embedding about 75 amino acid residues. A further characteristic of these domains is the localization of hydrophobic and/or aromatic residues in the loops connecting PG_II_ helices (Figure 3a). Given the properties of these PG_II_ helices, it is likely that they are functional to mediate interactions of PE_PGRS proteins with other proteins or non-proteinaceous components on the mycobacterial outer membrane. Along this line, we predict that these PG_II_ sandwiches structures are aligned orthogonally to the mycobacterial outer membrane (Figure 4); specificity of binding to a given target may be guided by the amino acids residing in the loops connecting the PG_II_ helices and that are exposed outward the mycobacterial cell. Conversely, the loops residues exposed inward are mainly hydrophobic and provide anchoring to the mycomembrane outer leaflet. In this scenario, the identified PG_II_ sandwich domains of PGRS portions would provide the structural units to expose unique amino acids in the PGRS region that mediate specific interactions with different molecules. This would also explain the peculiar function of each PE_PGRS protein.

The picture shows the hypothetical model, inferred from the experimental results gathered so far, on the localization of PE_PGRS proteins on the mycobacterial outer membrane. The model highlights the putative role of the PG_II_ sandwich domains (green) aligned orthogonally to the mycomembrane and the unique amino acids residues residing in the loops connecting the PG_II_ helices (violet). The unique C-terminal domain found downstream of the PGRS domain is shown in blue.

### Unique C-terminal domain

Most of PE_PGRS proteins show a short (5–20 aa) and unique amino acids sequence at the extreme protein C-terminal end. However, in nine PE_PGRSs (PE_PGRS3, −11, −16, −17, −18, −30, −35, −50 and −59) the unique C-terminal domain is larger and can reach ≈ 300 amino acids in length. Interestingly, PE_PGRS3 and PE_PGRS50 present a homologous, arginine-rich C-terminal domain. Similarly, the unique C-terminal domain of PE_PGRS30 is highly homologous to the C-terminal domain of the PE-unique protein encoded by Rv3812 [50–52]. It is likely that the genetic sequences coding for these protein domains were mobilized or duplicated following intragenomic rearrangements and selected when expressed downstream of a given PE or PE_PGRS protein, which warrants localization on the mycobacterial cell wall, where these unique domains can exert their function. This is like to what observed in other PE-unique proteins as LipY, whose C-terminal domain with lipase activity can be found downstream of a PE domain (in LipY), a PPE domain or a secretion signal peptide, depending on the mycobacterial species [34]. Recent findings obtained when expressing the *Mtb* LipY in *M. marinum* indicate that processing of the PE domain by the protease PecA does not affect lipase activity [40].

### L-GRPLI motif

The amino acid sequence that usually extends from position ≈ 90–92 to position 135–140 of the PE_PGRS proteins bridges the PE and PGRS domains (Figure 2a). The proximal part of this sequence starts downstream of the PE domain with an EAA-sequence, followed by a region with some highly conserved amino acids at certain positions (as Q at positions 99 and N at position 110) but with some degree of polymorphism (Supplementary Figure 1). This region is a sort of linker (l) between the PE domain and the GRPLI motif. The GRPLI motif classically stands on position 120–124 or 127–131 except for the PE_PGRS11, where the l sequence is unusually longer (GRPLI at position 159–163). The GRPLI sequence is highly conserved among all the PGRSs with very few exceptions, the most remarkable being the substitution of proline (P) with aspartic acid (D) in PE_PGRS11. The conservation of amino acids at specific positions in the consensus sequence clearly indicates a key role of this domain in PE_PGRS protein localization and function (Figure 4). Interestingly, four PE_PGRS proteins, which are encoded by adjacent genes, show a second GRPLI motif within the PGRS domain (PE_PGRS3 and −4, −9, and −10).

### Working model

The paucity of studies on *Mtb* involving PE_PGRSs have so far hampered the establishment of a working model that can identify the protein domains responsible for protein translocation and localization on the mycobacterial surface. Heterologous expression of PE_PGRSs in *M. smegmatis* demonstrated that PE_PGRS can reach the mycobacterial surface and that the PE/l-GRPLI domain is necessary and sufficient to warrant translocation of the whole protein on the mycobacterial surface despite the fact that the ESX-5 T7SS is missing [41,51,53]. It remains to be determined whether other ESX T7SS may compensate the lack of ESX-5 to warrant secretion/translocation of heterologously expressed PE_PGRSs in *M. smegmatis*. Indeed, expression in *M. smegmatis* or *M. bovis* BCG of a recombinant fusion protein expressing MPT64 downstream the PE/l-GRPLI domain of PE_PGRS33 resulted in the surface localization of MPT64, where it remained tightly associated with the mycobacterial cell wall [53,54]. As expected, point mutations affecting the highly conserved amino acids in the PE domain, or deletions of the PE domain, significantly affected PE_PGRS33 localization, further demonstrating the critical role of the PE domain in protein localization [43,55]. However, studies in *M. marinun* and in *Mtb* showed that PE_PGRS proteins are secreted in an ESX5-dependent mechanism [21,56,57]. PE_PGRSs were detected in the *M. marinum* culture supernatant by using an anti-PGRS monoclonal antibody or by specifically detecting PE_PGRS45, and secretion was abolished in the ESX-5 mutant [21]. More recently, deletion of the *ppe*38/*ppe*71 region, or natural mutations in this gene locus occurring in some *Mtb* Beijing strains and in *M. bovis* BCG, affected PE_PGRSs secretion in MTBC providing compelling evidences for the ESX5-dependent secretion of these proteins [57,58]. In a recent report, Burggraaf et al. [40] demonstrates that in *M. marinum* LipY and PE_PGRS proteins are processed by PecA, a PE_PGRS protein itself, that cleaves the PE domain on the mycobacterial surface. Interestingly, PecA can cleave the *Mtb* LipY (LipYtub) protein when heterologously expressed in *M. marinum*, suggesting that the same processing may occur in *Mtb*. While these studies significantly contributed to better understanding the mechanisms of PE_PGRS protein translocation in *Mtb* and the consequences in TB pathogenesis and *Mtb* virulence, many aspects remain obscure. For instance, the fact that certain PE_PGRS proteins can be extracted by using non-anionic detergents as Genapol [43,53] suggest their association with the mycobacterial cell wall. Similarly, the unique C-terminal domain of PE_PGRS30 (≈ 300 amino acids) seems not available on the mycobacterial surface but rather directed toward the periplasm [51], where it may be involved in the protein polar localization [59]. More recently, PecA, the *M. marinum* homologue of *Mtb* PE_PGRS35, could not be found in the culture supernatant despite being cleaved of the PE domain in the mycobacterial surface, pointing for an association with the mycobacterial outer membrane [40].

These findings support a model that contemplates the tight association of the PGRS domain with the mycomembrane (Figure 4). We hypothesize that PE_PGRS form homodimers and that the PE domain, following translocation/secretion through the inner bacterial membrane, remains anchored in the inner part of the mycomembrane or cleaved by proteases, as recently suggested [40]. Hence, PecA or similar proteases cleave the PE domain releasing the mature form of the PE_PGRS protein (that is the PGRS domain with the unique C-terminal domain) that remains associated with the mycomembrane. The l/GRPLI contains a transmembrane domain that position the PGRS on the mycobacterial surface. The GGA-GGX sequences are organized in PG_II_-like helices, closely spaced together to form flat multiple-layer domains [47,48] that extend as fibrils from the mycomembrane outer leaflet outwards. The unique amino acid sequences, intercalating the GGA-GGX repeats, would be positioned either on the external tip of these PG_II_ fibrils to interact with host components or other molecules, or inwards, to ensure proper embedding of the protein on the mycomembrane. Indeed, the presence of multiple hydrophobic phenylalanine-leucine/isoleucine residues in these intercalating sequences lend support to this hypothesis (Figure 3b and Supplementary Figure 2).

## Experimental evidences on PE_PGRSs

Since their identification [11], the role of PE_PGRS proteins in *Mtb* biology and TB pathogenesis raised several hypothesis and speculations, though the studies that attempted to investigate the function of these proteins are relatively limited. In this chapter, we summarize the knowledge and experimental evidences gathered so far on PE_PGRSs.

### PE_PGRS3 and PE_PGRS4

PE_PGRS3 (Rv0278c, 957 aa) and PE_PGRS4 (Rv0279c, 837 aa), are two highly homologous PE_PGRSs. PE_PGRS3 presents a unique and peculiar arginine‐rich C‐terminal domain (≈ 80 aa) which is not present in PE_PGRS4. The highly homologous PGRS domain of the two proteins presents an extra GRPLI motif at position 528–532 and 421–425, for the PE_PGRS3 and PE_PGRS4, respectively.

The presence of these two neighboring, highly homologous genes is a classic example of a gene duplication event, which is a hallmark of other *pe_pgrs* [28,60]. Indeed, the Rv0278c genetic locus, but not Rv0279c, is a recombination hot spot subjected to genomic rearrangements especially in some *Mtb* lineages [60]. The fact that Rv0278c gene is duplicated in *Mycobacterium canetti* and *Mycobacterium bovis*, but not in *Mtb* strains, is somehow intriguing.

Interestingly, these two genes were shown to be differentially regulated, with *pe_pgrs*4 constitutively expressed and *pe_pgrs*3 specifically expressed following long-term exposure to low inorganic phosphate concentrations [61], underscoring the evolutionary divergence observed in these two genes in MTBC. Heterologous expression of PE_PGRS3 in *M. smegmatis* demonstrated that the arginine-rich domain is available on the mycobacterial surface, can significantly affect net surface charge and can promote adhesion to host cells and tissues [61]. While more studies in *Mtb* are required to better characterize the role of PE_PGRS3 in TB pathogenesis, it is tempting to speculate that the peculiar expression pattern and unique arginine-rich domain may directly implicate PE_PGRS3 during the persistence of *Mtb* in low phosphate environments as macrophages and granulomas.

### PE_PGRS5

PE_PGRS5 (Rv0297) is a 591 amino acids protein which shows a very high similarity with the N-terminal domain of PE_PGRS33, though a substantial variation is observed between the amino acid sequences in the PGRS domain. Furthermore, *in silico* analysis of the PE_PGRS5 predicted the presence of an extended disordered region within the PGRS domain containing seven endoplasmic reticulum signal peptides [62]. Indeed, PE_PGRS5 was demonstrated to localize with the endoplasmic reticulum in eukaryotic cells transfected with a plasmid expressing PE_PGRS5 and this cell localization was not dependent on the protein N-terminal domain (PE domain) [62]. Interestingly, expression of the sole PGRS domain of PE_PGRS5 was sufficient to activate the macrophage unfolded-protein-response pathway and to produce endoplasmic reticulum stress markers through the TLR-4 activation, which in turn promoted alteration of the intracellular calcium homeostasis, increase in NO and ROS production and caspase 8-mediated apoptosis [62]. It can be therefore hypothesized that release of PE_PGRS5, or of its PGRS C-terminal domain, from the mycobacterial cell surface by *Mtb* residing in infected host cells, may lead to translocation of the protein in the endoplasmic reticulum and activation of specific host pathways. However, proper localization of PE_PGRS5 and other PE_PGRS proteins in macrophages or other *Mtb* infected host cells is required to support this hypothesis.

### PE_PGRS11

PE_PGRS11 (Rv0754) is a 584 amino acids protein with a very short PGRS domain (≈100 amino acids over the ≈ 420 amino acids that make the C-terminal domain). Interestingly, the amino acid sequence that extends from the end of the PE domain to the GRPLI motif (the linker domain l) is longer than what observed in most PE_PGRSs and a Pro→Glu substitution is present in the GRPLI (Supplementary Figure 1). The unique C-terminal domain contains a fully functional phosphoglycerate mutase domain, as shown by testing the enzymatic activity of the recombinant PE_PGRS11 protein [63]. Overexpression of PE_PGRS11 in *M. smegmatis* enhanced resistance to H_2_O_2_-induced oxidative stresses in infected lung epithelial cells, which was dependent upon the enzymatic activity of the phosphoglycerate mutase domain. Interestingly, interaction of PE_PGRS11 with TLR2 triggered COX-2 and Bcl2 expression in infected cells, with these anti-apoptotic signals mediating resistance to oxidative stresses. Moreover, expression of the *pe_pgrs*11 gene was found to be up-regulated in hypoxic conditions, which are thought to occur within granulomas [63,64]. The availability of PE_PGRS11 on the *Mtb* surface and its expression profile, prompted to hypothesize that PE_PGRS11 can interact with host components and contribute to allow evasion of *Mtb* from oxidative stresses [63]. PE_PGRS11 can also mediate the activation of dendritic cells in a TLR2-dependent mechanism that promotes the secretion of pro-inflammatory cytokines [65]. While not directly demonstrated, it is likely that the small PGRS domain might be responsible for this activity.

### PE_PGRS16

PE_PGRS16 (Rv0977) is a 923 amino acids protein characterized by a large C-terminal domain of 273 amino acids downstream of the PGRS domain. Particularly, the unique C-terminus presents a marked hydrophobicity as assessed by ≈ 88% of not polar (45.5%) and polar, but not charged amino acid residues (42.8%). The structural characterization of the unique C-terminal domain demonstrated the presence of an aspartic proteinase-like domain [66]. Despite the presence of DTG/DSG amino acidic motifs, which are classically found in the aspartic proteinases such pepsins, PE_PGRS16 seems to lack proteolytic activity probably because of substantial differences in the substrate binding sites that could require alternative substrates or environmental conditions with respect to common pepsin substrates [66].

Intriguingly, *pe_pgrs*16 was upregulated under nutrient-depleted growth conditions, in bone marrow infected macrophages and finally in aerogenically infected mice [67]. Although we do not know the PE_PGRS16 biological role, the observed transcriptional profile of its structural gene may suggest its involvement in the late phase of the infection.

### PE_PGRS17 and PE_PGRS18

PE_PGRS17 (Rv0978c) and PE_PGRS18 (Rv0980c) are 331 and 457 amino acids proteins, respectively, with a large and unique C-terminal domain. The two genes share a high degree of homology, suggesting the occurrence of an intra-genomic duplication event [23]. Indeed, *pe_pgrs*17 is a recombination hot-spot site [60].

The unique C-terminal domains of these two PE_PGRSs share a very high homology (≈ 90%) and blast analysis reveals that this domain has a high similarity with the YncE family of proteins described to play a role in bacterial survival of *Salmonella enterica* serotype Typhi [68]. The only available experimental evidences come from heterologous expression of these proteins in *M. smegmatis*. PE_PGRS17 binds to TLR2 and activate the ERK1/2, p38 MAPK and NF-κB signaling pathway, promoting TNF-α secretion [65,69]. PE_PGRS18 promotes apoptosis of infected macrophages by inhibiting cytokines as IL-6, IL-1β, and IL-10 and inducing secretion of IL-12 [70]. Heterologous expression of both proteins in *M. smegmatis* promoted cell death and enhanced intracellular mycobacterial survival over parental *M. smegmatis* strains, similarly to what observed for other PE_PGRSs. However, the role and contribution of the unique C-terminal domain in this process is still unclear.

### PE_PGRS26

PE_PGRS26 (Rv1441c) is 491 amino acids protein with the typical PGRS domain. Contrary to other *pe_pgrs* genes, transcriptional analysis of *pe_pgrs*26 indicates its downregulation in *Mtb* infecting macrophages or during the chronic-persistent phase of infection in mice [67]. Accordingly, mice infected with a *Mtb*Δ*pe_pgrs*26 mutant showed an attenuated phenotype during the acute phase of infection, but virulence was rescued during chronic/persistent phase (70 day post-infection), suggesting that this protein may be necessary during the acute phase [50,67].

### PE_PGRS29

PE_PGRS29 (Rv1468) is a 370 amino acids protein with a short C-terminal domain (≈ 11 amino acidic residues) and the classical l-GRPLI motif with an unusual substitution of the glycine with the polar amino acid asparagine. Chai et al. [71] identified a eukaryotic-like ubiquitin-associated (UBA) domain in the PE domain (position 32–66). In a series of elegant experiments, the authors demonstrated that during *Mtb* infection of macrophages, poly-ubiquitin chains bind to PE_PGRS29 available on the mycobacterial surface in an E3 ligases-independent manner and that *Mtb* ubiquitination occurred either in the permeable *Mtb*-containing phagosome or in the bacilli surviving in the cytosol. Interestingly, the *Mtb*Δ*pe_pgrs*29 mutant showed enhanced survival in infected macrophages compared to the parental *Mtb* strain, indicating that PE_PGRS29-dependent ubiquitin targeting of mycobacteria is crucial for the *Mtb* autophagic clearance. Moreover, lack of PE_PGRS29 abolished binding of particles containing the autophagosomal – associated protein LC3 and resulted in the accumulation of bacilli in the cytosol, suggesting that PE_PGRS29 is important for the autophagic clearance of cytosolic *Mtb. In vivo* experiments confirmed the enhanced virulence of the *Mtb*Δ*pe_pgrs*29 over the parental *Mtb* strain, with the former showing enhanced bacterial loads, inflammation and tissue damage. These results suggest that the PE_PGRS29-ubiquitin interaction mediating autophagic clearance of *Mtb* may be a smart strategy deployed by *Mtb* to achieve long-term intracellular survival in infected macrophages while avoiding excessive and potentially deleterious inflammatory responses.

### PE_PGRS30

PE_PGRS30 (Rv1651c) is a 1011 amino acid protein which presents a unique and large C-terminal domain of 306 amino acids. Characteristically, PE_PGRS30 shows a high homology with the MAG24 protein of *M. marinum*, which is specifically upregulated in granulomas [72]. Furthermore, the unique C-terminal domain of PE_PGRS30 shows a high homology with the C-terminal domain of the protein encoded by Rv3812 [52], a PE-unique protein erroneously included in the PE_PGRS despite the lack of the PGRS and l-GRPLI domains (formerly PE_PGRS62 that we have now renamed PE39) [16]. The *pe_pgrs*30 gene is upregulated during growth in infected macrophages and in murine host tissues during the chronic persistent phase of infection [73]. In line with these findings, the *Mtb*Δ*pe_pgrs*30 mutant shows an attenuated phenotype in infected mice, mainly during the chronic phase of infection, with a dramatic drop in bacilli in the lung tissue and a remarkable reduction in tissue damage compared to the parental strain [50]. Interestingly, complementation of the *Mtb*Δ*pe_pgrs*30 with a plasmid expressing a functional deletion mutant of PE_PGRS30 missing the C-terminal domain, fully restored the mutant virulence, pointing to the key role of the PGRS domain [50]. Moreover, *in vitro* experiments carried out in macrophages confirmed that PE_PGRS30 is required to block phagosome maturation by *Mtb* and again that the unique C-terminal domain is dispensable for this process [50]. Hence, PE_PGRS30 is required for the full virulence of *Mtb* and as such can be considered a virulence factor, although the exact mechanism involved in this process remains to be determined. There are indications that the C-terminal domain of this protein is not available on the surface [51], while the PGRS domain was shown to mediate protein localization [59] and may well interact with host components and exert its role in *Mtb* pathogenesis.

### PE_PGRS33

PE_PGRS33 (Rv1818c) is a 498 amino acids protein and it is the first and probably the most studied protein of the family. It is a classical PE_PGRS protein with a short (≈ 10 amino acids) unique C-terminal domain. Several studies indicated that the *pe_pgrs*33 gene is constitutively expressed, with the level of transcripts detectable and similar in axenic culture, during infection of macrophages and in infected host tissues [67,73–75]. Several studies indicate that PE_PGRS33, similarly to other PE_PGRSs, is the target of the host humoral response during *Mtb* infection, although the multiple repeats and redundancy of the PGRS sequence, which is the domain recognized by the host antibodies, makes it difficult to assess the specificity of this response [45,76,77].

Heterologous expression of PE_PGRS33 in *M. smegmatis* promoted cell death and increased mycobacterial survival in macrophages and in intraperitoneally infected mice over the parental strain or the *M. smegmatis* recombinant strain expressing only the PE domain of PE_PGRS33, pointing for the key role of the PGRS domain in this process [42,78,79]. PE_PGRS33 triggered TNF-α and IL-12 secretion promoting cell necrosis and inflammation, as highlighted by the enlarged spleens of mice infected with *M. smegmatis* expressing PE_PGRS33 compared to controls [55,78,80,81]. However, experiments carried out with the purified recombinant protein or in eukaryotic cells transfected with a plasmid expressing PE_PGRS33, while confirming the ability of PE_PGRS33 to promote cell death implicated a mechanism involving apoptosis rather than necrosis [42,79]. Of note, PE_PGRS33 was able to interact with TLR2 to promote cell death and inflammation [42] and proper localization of PE_PGRS33 on the mycobacterial surface is required to activate the TLR2 pathway [55]. Moreover, an antiserum directed against the native form of PE_PGRS33 was able to abolish the secretion of TNF-α following infection of macrophages with *M. smegmatis* expressing PE_PGRS33, further supporting the key role of PE_PGRS33-TLR2 interaction [81]. In line with these findings, the *Mtb*Δ*pe_pgrs*33 mutant was impaired, compared to the parental strain, in its ability to enter in macrophages, but not epithelial cells, in a process involving activation of the TLR2-CR3 pathway, which activates the inside-out-signaling to promote *Mtb* entry in macrophages [44]. Interestingly, experiments carried out with *Mtb*Δ*pe_pgrs*33 complemented with a series of functional deletion mutants missing different portions of the PGRS domain suggest that the proximal PGRS domain (amino acid sequence encompassing positions 140–260) is sufficient to activate TLR2 [44].

The role of PE_PGRS33 in TB pathogenesis has been further investigated in *in vivo* experiments in mice, which rather than showing attenuation of the mutant compared to the parental strain showed an enhanced virulence during the chronic steps of the infection [31]. Similarly, the *Mtb*Δ*pe_pgrs*33 complemented with a naturally occurring frameshifted *pe_pgrs*33 allele, obtained from an *Mtb* strain belonging to an ancient lineage, caused enhanced tissue damage during the chronic steps of infection [31]. Genetic polymorphism analysis of the *pe_pgrs*33 in a collection of *Mtb* strains representative of the different phylogeographic lineages highlighted that this gene was under purifying selection, confirming other findings on other *pe_pgrs* genes [30]. Previous studies characterized naturally occurring *pe_pgrs*33 polymorphisms in *Mtb* clinical isolates, showing that large in-frame indels and frameshift mutations correlated with the absence of cavitation in lungs [82] or with TB meningitis in children [83]. Since these extra-pulmonary forms of TB are usually associated with extensive tissue damage and severe clinical patterns, it follows that the observed large genetic polymorphisms in *pe_pgrs*33 do not affect *Mtb* virulence [31]. The finding that *pe_pgrs*33 is missing in *M. marinum* and smooth tubercle bacilli [10] and it is under purifying selection in *Mtb* suggests that MTBC acquired *pe_pgrs*33 during the evolution to promote tissue damage and persistence in the lung tissue. Indeed, we hypothesize that it may play a critical role in the successful transmission of *Mtb* in humans [31].

### PE_PGRS35

PE_PGRS35 (Rv1983) is a 558 amino acids protein that contains a large unique C-terminal domain that shows 43,9% identity with the C-terminal domain of the PE_PGRS16 and comprises an aspartic proteinase domain. In a very recent report, Burggraaf et al. [40] showed that the *M. marinum* homolog of the PE_PGRS35 (MMAR_2933) can process the *Mtb* protein LipY (LipY_tub_) when expressed as heterologous protein in *M. marinum*. Processing of LipY_tub_ by the *M. marinum* PE_PGRS35 homologue occurs at multiple sites, near or within the YxxxD/E secretion motif within the PE domain or in the linker domain of LipY, in line with previous findings [34]. Since the protease activity of MMAR_2933 results in the cleavage of the PE domain, the PE_PGRS35 homologue has been renamed PecA (PE cleavage protein A) [40]. Interestingly, PecA is not secreted in the culture medium by *M. marinum* and cleavage occurs at the mycobacterial cell surface where the protease can cleave itself and other PE_PGRS proteins. It remains to be determined the role of PecA (PE_PGRS35) in *Mtb*.

### PE_PGRS41

PE_PGRS41 (Rv2396) is a small PE_PGRS protein of 361 amino acids. Like PE_PGRS11, PE_PGRS41 has a longer l-GRPLI domain compared to most PE_PGRSs. Interestingly, *pe_pgrs*41 belongs to the *apr*ABC gene locus that includes the two upstream genes (*apr*A and *apr*B), with *apr*C corresponding to *pe_pgrs*41. The two-component regulator *pho*PR senses the acidic pH of the phagosome and induces expression of the *apr*ABC genes [84]. These results suggest the implication of *apr*ABC, which is unique to MTBC, in the mechanism that grants adaptation of *Mtb* to intracellular lifestyle. However, expression levels of the *apr*C/*pe_pgrs*41 genes are much lower and poorly modulated compared to what observed for the *apr*AB genes. Moreover, the functional relation between the proteins encoded by the *apr*ABC locus remains undisclosed. In line with these findings, heterologous expression of PE_PGRS41 in *M. smegmatis* enhanced mycobacterial survival in macrophages thanks to the inhibition of autophagy, increased cytotoxicity, and dampening of inflammatory responses [85]. Hence, PE_PGRS41 can be included in the list of MTBC virulence factors known to play an important role in TB pathogenesis [85].

### PE_PGRS47

PE_PGRS47 (Rv2741) is a 525 amino acid protein that recent studies implicated in *Mtb* pathogenesis. The *Mtb*Δ*pe_pgrs*47 mutant is impaired in its ability to replicate intracellularly in macrophages or to persist in host tissues at late stages of *Mtb* infection in mice [86], similarly to what seen for the *Mtb*Δ*pe_pgrs*30 mutant [50]. Interestingly, PE_PGRS47 can inhibit bacterial-derived antigen processing and presentation through the MHC-II pathway, which lead to reduced CD4 T cell responses against heterologous *Mtb* antigens as TB9.8 and Ag85B at the early and chronic-persistent phase of infection [86]. Interestingly, the finding that PE_PGRS47 can also block autophagy and phagosomes acidification in *Mtb* infected macrophages provides a potential mechanism for the observed inhibition of antigen processing and presentation [86]. These recent results are of interest and experimentally support the hypothesis that PE_PGRS may be involved in the immune evasion strategies [11,12].

## PE_PGRS in TB pathogenesis

Despite the relative high homology in amino acid sequence, structural organization, and the repetitive and apparently redundant PGRS domain, the experimental evidences collected so far indicate that different PE_PGRS proteins can play distinct roles in *Mtb* biology. Studies that assessed the transcriptional expression profile of *pe_pgrs* genes demonstrated that some genes were specifically expressed or up-regulated under certain environmental conditions (low pH, nutrient starvation, etc.) or in *Mtb* residing inside macrophages, and that level of expression of different *pe_pgrs* genes could vary significantly [41,61,67]. For instance, *Mtb* constitutively expresses *pe_pgrs*33 while *pe_pgrs*30 transcription is specifically activated in *Mtb*-infected macrophages [41,50]. These findings are in line with the fact that *pe_pgrs* genes are scattered in the genome where they are organized in single open reading frames, similarly to *ppe_mptr* genes [87], but different from *pe*/*ppe* couplets, whose genes are co-expressed and the corresponding proteins form dimers and are functionally linked [11,33,88]. These observations, and a body of other experimental evidences, indicate the expression of PE_PGRS proteins as single polypeptides and suggest that each protein can exert its function without a specific protein partner. Moreover, the finding that *pe_pgrs* genes are in purifying selection in *Mtb* [30] tend to exclude the functional redundancy of PE_PGRS proteins in *Mtb* biology, rather suggesting that each protein may exert a unique function.

**Figure 5.**
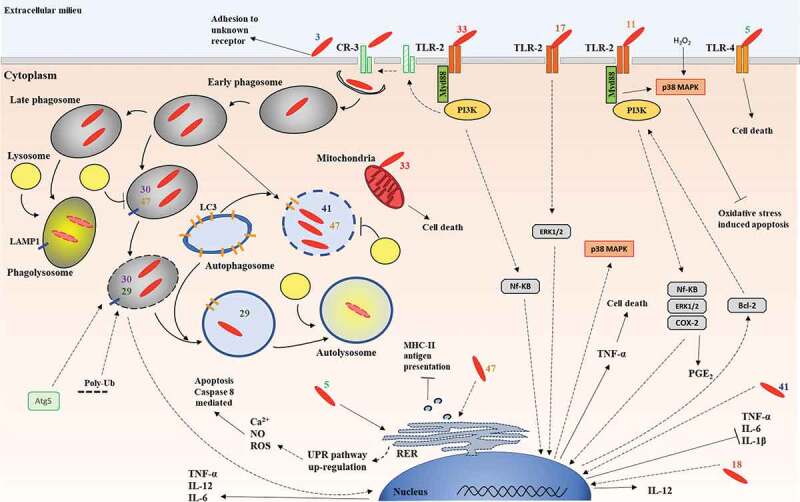
Schematic representation of the pathogenetic processes at cellular level involving PE_PGRS proteins during *Mtb* pathogenesis.

Since the identification of PE_PGRS proteins in *Mtb* [11] several studies implicated PE_PGRS proteins in different steps of TB pathogenesis (Figure 5 and Table 1).

**Table 1.** Schematic summary of the PE_PGRS proteins functional activities during *Mtb* pathogenesis.

Name	Length (aa)	Proposed functional role in Mtb^a^	References^b^
PE_PGRS3	957	Promotes adhesion to macrophages and alveolar epithelial cells; Increases persistence in host tissues;	^61^
PE_PGRS5	591	Induces caspase 8 mediated apoptosis UPR/TLR-4 dependent;	^62^
PE_PGRS11	584	Enhances resistance to H_2_O_2_-induced oxidative stresses thanks to the anti-apoptotic signals triggered by the TLR2-dependent activation of COX-2/Bcl2 expression;	^63^
PE_PGRS17	331	Promotes TNF-α secretion via ERK1/2 – p38 MAPK – NF-κB signaling pathway via TLR-2;	^65, 69^
PE_PGRS18	457	Promotes apoptosis; Induces IL-12 and inhibits IL-6, IL- 1β and IL-10;	^70^
PE_PGRS29	370	Induces autophagic clearance of cytosolic *Mtb* by promoting binding to poly-ubiquitin;	^71^
PE_PGRS30	1011	Blocks phagosome maturation to enhance *Mtb* intracellular survival;	^50^
PE_PGRS33	498	Induces cell death and inflammation in a TLR-2 dependent mechanism; Promotes entry in macrophages via the TLR-2/CR-3 inside-out-signaling pathway; Promotes damage and persistence in lung tissue;	^31, 42, 44, 45, 55, 78, 79^
PE_PGRS41	361	Inhibits autophagy; Blocks TNF-α, IL-12 and IL-6 secretion;	^84, 85^
PE_PGRS47	525	Blocks autophagy and phagosome acidification; Inhibits MHC-II antigen presentation which suppresses *Mtb*-specific CD4 + T cell responses;	^86^

^a^The functional role of the PE_PGRS proteins indicated in the table has been proposed based on experimental evidences gathered in different experimental models as outlined in the text. Not all the experimental evidences have been collected using *Mtb* as a model. ^b^The numbers correspond to what indicated in the reference list section

The figure identifies the extracellular and intracellular steps in *Mtb* pathogenesis for which the involvement of a PE_PGRS proteins has been demonstrated. The number close to the bacilli (shown in red) correspond to the PE_PGRS (for example, 33 indicates interaction of PE_PGRS with TLR2).

PE_PGRS3 and PE_PGRS33 promote *Mtb* entry in host cells: PE_PGRS3, by promoting adhesion to macrophages and epithelial cells through the “sticky” arginine-rich C-terminal domain [61]; PE_PGRS33, specifically targeting TLR2, that by activating the inside-out signaling stimulates bacterial entry through the CR3 receptor [44], which warrants enhanced bacterial survival and virulence [89]. It remains to be determined whether other PE_PGRS proteins that present an arginine-rich C-terminal domain (as PE_PGRS50 and −55) or that interact with TLR2 (as PE_PGRS11 and −17) contribute to *Mtb* entry in host cells.

Following entry in macrophages and engulfment in phagosomes, *Mtb* deploys a sophisticated arsenal of protein and effector molecules to prevent phagosome-lysosome fusion and evade killing [90]. Among the most important effectors are the proteins secreted by the T7SS of the ESX family, that in *Mtb* are present in five copies [91]. ESX-1 is the best characterized of the five T7SS and its inactivation in the vaccine strain *Mycobacterium bovis* Bacille Calmette and Guerin (BCG) is the main cause of attenuation [92]. *Mtb* survival and replication in macrophages requires secretion through the ESX-1 system of ESXA/B and other proteins that permeabilize the phagosome membranes to promote access of *Mtb* proteins to the cytosol and prompt bacilli translocation in the cytoplasm [93,94]. Among the proteins that contribute to inhibit phagosome lysosome fusion is PE_PGRS30 that is specifically expressed by *Mtb* intracellularly and prevents phagosome acidification in a yet unknown mechanisms, thereby enhancing the survival of *Mtb* in macrophages [50,95]. PE_PGRS47 is another protein involved in these events by inhibiting phagosome acidification and antigen processing [86]. While some PE_PGRSs seem to enhance *Mtb* survival and virulence in macrophages, others PE_PGRSs seem to counterbalance these processes by modulating autophagy. Autophagy can support bacterial clearance in macrophages, either by engulfing phagosomes-containing bacteria (xenophagy) or by targeting cytosolic bacteria with ubiquitin and LC3 [71]. PE_PGRS29 recruits ubiquitin on cytosolic *Mtb*, or bacilli otherwise accessible to ubiquitin in the permeabilized phagosome, to trigger host xenophagy and promote bacterial clearance, apparently to reduce inflammatory responses [71]. Conversely, PE_PGRS47 suppresses autophagy in *Mtb* infected macrophages with important consequences not only in bacterial survival but also in antigen presentation of key *Mtb* antigens [86] and PE_PGRS41 inhibits autophagy when heterologously expressed in *M. smegmatis* [70]. The current experimental evidences indicate that several PE_PGRS proteins interfere or modulate host autophagy in macrophages with important consequences on the *Mtb* intracellular survival, antigen presentation and associated inflammatory responses.

The ability to promote inflammatory responses, oxidative stresses and cell death primarily by cell necrosis has been demonstrated for PE_PGRS11, −17, −18, −30 and −33. Usually, PE_PGRSs can trigger the secretion of inflammatory cytokines as TNF-α and IL-12 by binding to TLR2 and activation of the downstream signaling cascade [42,55,78–81] or by directly interacting intracellularly with mitochondria or other organelles [96]. The associated cell death further amplifies the inflammatory responses, thereby contributing to the classical necrotic tissue damage that is a hallmark of TB disease.

**Figure 6.**
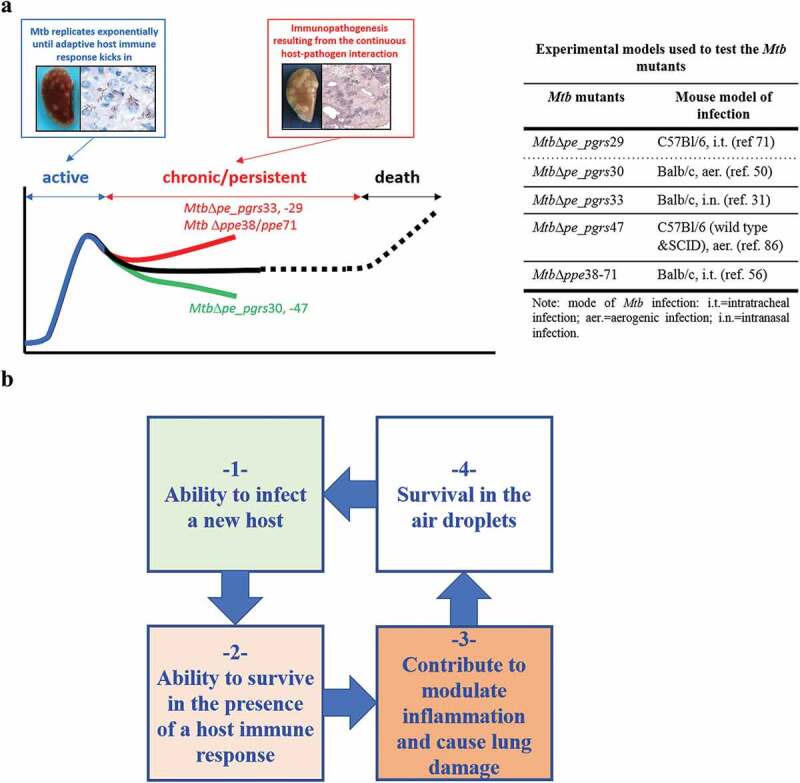
Role of PE_PGRS proteins in TB pathogenesis.

The consequences of these events taken place at cellular level have been investigated in *in vivo* models of infection and the few results available highlight a complex picture that again prevents the disclosure of a common and overarching mechanism for the role of PE_PGRS in TB pathogenesis. PE_PGRS proteins are among the most abundant *Mtb* proteins in granulomas during acute and chronic TB disease in guinea pigs [97], and *pe_pgrs* genes are differentially regulated in host tissues during *Mtb* infection [73,80]. Inactivation of some *pe_pgrs* genes resulted in an attenuated phenotype *in vivo* when immunocompetent mice were infected as for the *Mtb*Δ*pe_pgrs*30 [50] and *Mtb*Δ*pe_pgrs*47 [86] mutants, while the *Mtb*Δ*pe_pgrs*33 [31] and the *Mtb*Δ*pe_pgrs*29 [71] mutants showed a hypervirulent phenotype compared with the parental control. While inactivation of *pe_pgrs* genes may lead to different and apparently conflicting results, a key and common feature of these *in vivo* experiments is the emergence of a clear phenotype in the *Mtb* mutant during the chronic persistent steps of infection (Figure 6a). Interestingly, the inactivation of some PE_PGRS proteins has a significant impact primarily in the histopathological features of the lung lesions, with a dramatic increase in tissue damage and inflammation associated with the inactivation of PE_PGRS29 and PE_PGRS33, or with a significant reduction in tissue damage as for PE_PGRS30 and PE_PGRS47. It would be of interest to investigate these *Mtb pe_pgrs* mutants in animal models of infection that better mimic human TB, as aerogenic infection of guinea pigs, rabbits, or non-human primate.

(a) Mutation of some *pe_pgrs* genes induce a clear phenotype during the chronic persistent steps of the infectious process. The experimental conditions and animal models of infection used in these experiments are reported on the right side of the panel. (b) Schematic with the key steps in the natural history of TB in immunocompetent humans. PE_PGRS proteins play a key role in steps 2 and 3, when the interplay between *Mtb* and the complex immune system of mammals occurs.

In line with these findings, inactivation of the *ppe*38/*ppe*71 gene locus abolished secretion of PE_PGRS and PPE_MPTR proteins in *Mtb*, resulting in an *Mtb* strain with enhanced virulence, at least in mice [56]. Interestingly, the phenotype of the *Mtb*Δ*ppe*38/*ppe*71 mutant and parental *Mtb* CDC1551 strain diverged primarily during the chronic/persistent steps of the infectious process in mice, with a significant impact on lung bacterial burden and most importantly tissue damage in the lung parenchyma [56]. These findings suggest at least two important considerations. First, that the ability of PE_PGRS proteins to promote inflammatory processes, or to modulate cell death and manipulate autophagy, can have relevant consequences at tissue level; second, that these events require or are amplified by the presence of the adaptive immune response that is the hallmark of the chronic persistent steps of *Mtb* pathogenesis. The impact that genetic polymorphisms of the *pe_pgrs*33 have on the clinical outcome of TB disease [83,98] and on *Mtb* virulence as assessed in the experimental mouse model of TB, suggest that at least some PE_PGRS proteins can exert important immunomodulatory activities in TB pathogenesis. It has been hypothesized that the strong selective pressure on *pe_pgrs*33 combined with its role in TB pathogenesis indicate its involvement in TB transmission [31]. Moreover, these immunomodulatory properties of PE_PGRS may on the other hand mitigate *Mtb* virulence in the host tissue to permit prolonged survival in the infected host [56].

Although more experimental evidences are needed to elucidate the role of PE_PGRS proteins in TB pathogenesis, the current body of evidences suggest that these proteins may have emerged and evolved in MTBC to resist, modulate or manipulate the complex mammal host immune system. In line with this hypothesis, PE_PGRSs are expected to contribute significantly to the main abilities that make *Mtb* one of the most successful human pathogens. We suggest their involvement primarily in supporting the ability of the tubercle bacilli to survive in the presence of a strong immune response and in modulating the complex inflammatory processes that shape the dynamic host–microbe interaction that can lead to active disease, primarily in the lung tissue, where the extensive tissue damage is instrumental for *Mtb* transmission (Figure 6b).

## Closing remarks

The emergence of *Mtb* as a human pathogen, whose survival is dependent upon the ability of the bacilli to spread from a patient with active pulmonary TB to a naïve host, was accompanied by significant genetic changes compared to the environmental mycobacterial progenitors. Among the most relevant are the switch of *Mtb* into a monomorphic bacterium, the marked reduction in genome size accompanied by the expansion and diversification of genes belonging to the *pe* and *ppe* families. The set of *pe_pgrs* genes in *Mtb* are overall genetically well conserved and in positive selection [30], suggesting a role in the *Mtb* biology different from that proposed earlier which implicated PE_PGRS proteins in antigenic variability as a source for immune evasion strategies [11]. In this systematic review, we summarized the actual knowledge on this important and enigmatic family of proteins and proposed some original hypothesis on the structure/function relationship of PE_PGRS protein domains. We highlight the fact that the PE domains of PE_PGRS protein paralogs appear to be more structurally conserved than the PE domain of PE/PPE and PE unique proteins and speculate, based also on modeling indicating that the PE domain cannot be stable on its own that PE_PGRSs exist as homodimers. We also propose for the first time a model where the repetitive GGA-GGX amino acid motifs found in the PGRS domain are organized in structural units based on PG_II_ sandwich modules. These PG_II_ units are instrumental for the PGRS anchoring to the mycomembrane outer leaflet while allowing proper exposure of unique amino acids found on the opposite side of the sandwich outward, where they are available for interaction with host components. From this perspective, the variable sequences present in between GGA-GGX conserved spacers would contribute either to the anchoring of the protein to the cell or to its specific function, depending on the side of the sandwich in which they are exposed. The large variability of these variable sequences among PE_PGRS proteins might help to explain why proteins with so similar structural features have so different roles in *Mtb* physiology.

## Supplementary Material

Supplemental Material
