# Supplementary material for: PE_PGRS proteins of Mycobacterium tuberculosis: A specialized molecular task force at the forefront of host–pathogen interaction
Source: Virulence. 2020 Jul 25;11(1):898–915. doi: 10.1080/21505594.2020.1785815 (PMC7550000; doi:10.1080/21505594.2020.1785815)
Supplement: Supplemental Material [file KVIR_A_1785815_SM4297.zip › Supplementary Figure caption.docx]

**Supplementary Figure 1. The linker domain (l) of the PE_PGRSs shows conserved features.** Alignment of the linker amino acids interspersed between PE and PGRS, that extends from position ≈ 90-92 to position 135-140 of the PE_PGRS, show that besides the GRPLI motif also other amino acids are commonly conserved. The proximal part of this sequence usually starts with an EAA- sequence, followed by a region with some highly conserved amino acids at certain positions, as glutamine (Q) at positions 99 and asparagine (N) at position 110, but with some degree of polymorphism. GRPLI motif stands on position 120 – 124 or 127 – 131 except for the PE_PGRS11, where the linker sequence is unusually longer (GRPLI at position 159 – 163) and GRPLI shows the substitution of proline (P) with aspartic acid (D) in PE_PGRS11. Prediction of the spatial conformation of some linker domain highlight not a defined structure and probably not like other characterized proteins. GRPLI motif could induce a change in PE_PGRS direction but further studies are needed. Multiple alignment was performed by using *Clustal Omega* (*https://www.ebi.ac.uk/Tools/msa/clustalo/*) online software and consensus pattern definitions are based on the classes equivalence, set on the aminoacidic residues that share some physicochemical property.

Spatial organization was obtained by using *Protein Homology/analogY Recognition Engine V 2.0* (Phyre^2^) (*http://www.sbg.bio.ic.ac.uk/phyre2/html/page.cgi?id=index*) and the prediction result visualized with EzMol^1.3^ (*http://www.sbg.bio.ic.ac.uk/~ezmol/*).
