# Supplementary figures and images for: PE_PGRS proteins of Mycobacterium tuberculosis: A specialized molecular task force at the forefront of host–pathogen interaction
Source: Virulence. 2020 Jul 25;11(1):898–915. doi: 10.1080/21505594.2020.1785815 (PMC7550000; doi:10.1080/21505594.2020.1785815)

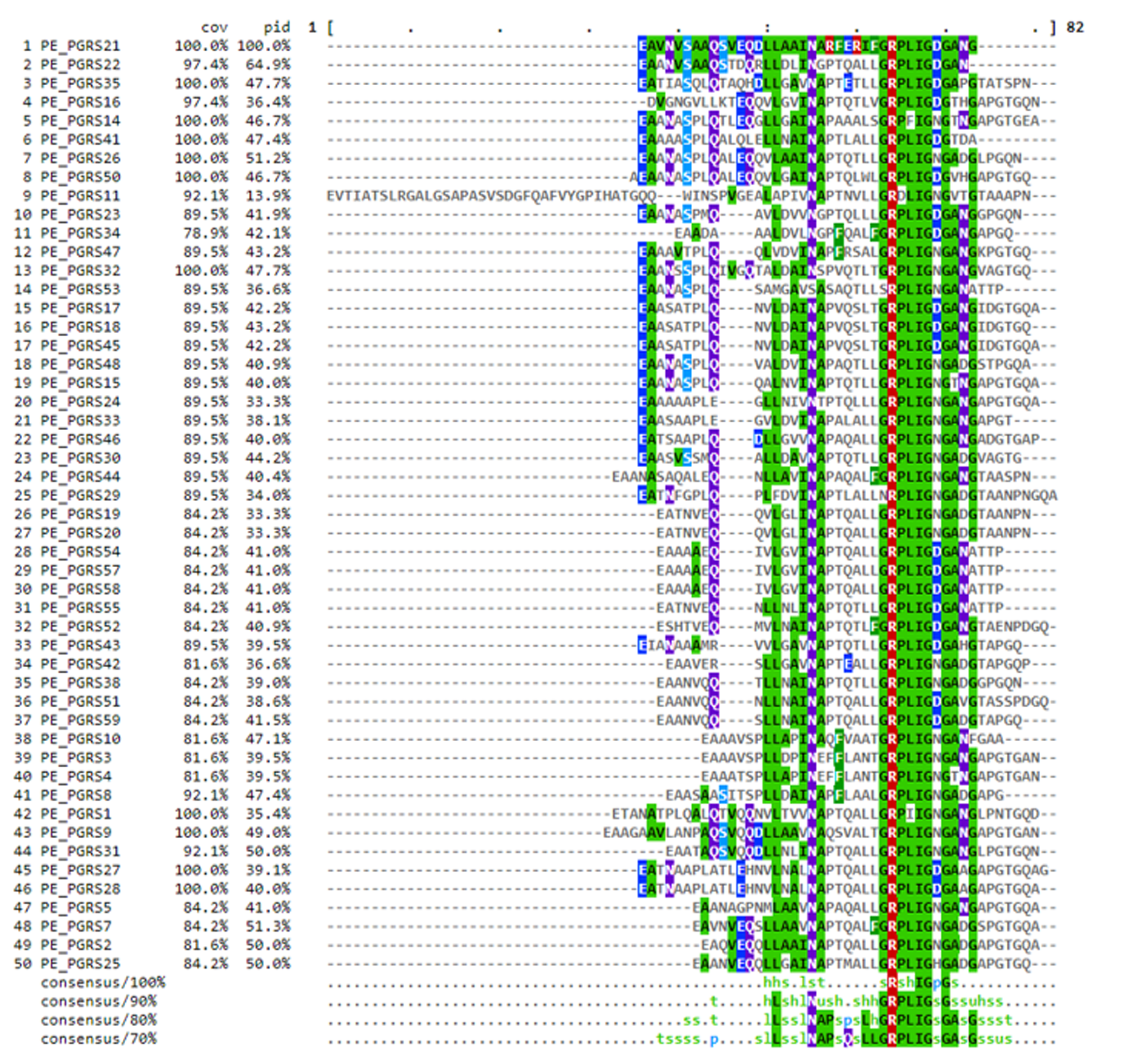

Supplement: Supplemental Material [file KVIR_A_1785815_SM4297.zip › figure S1.tif]

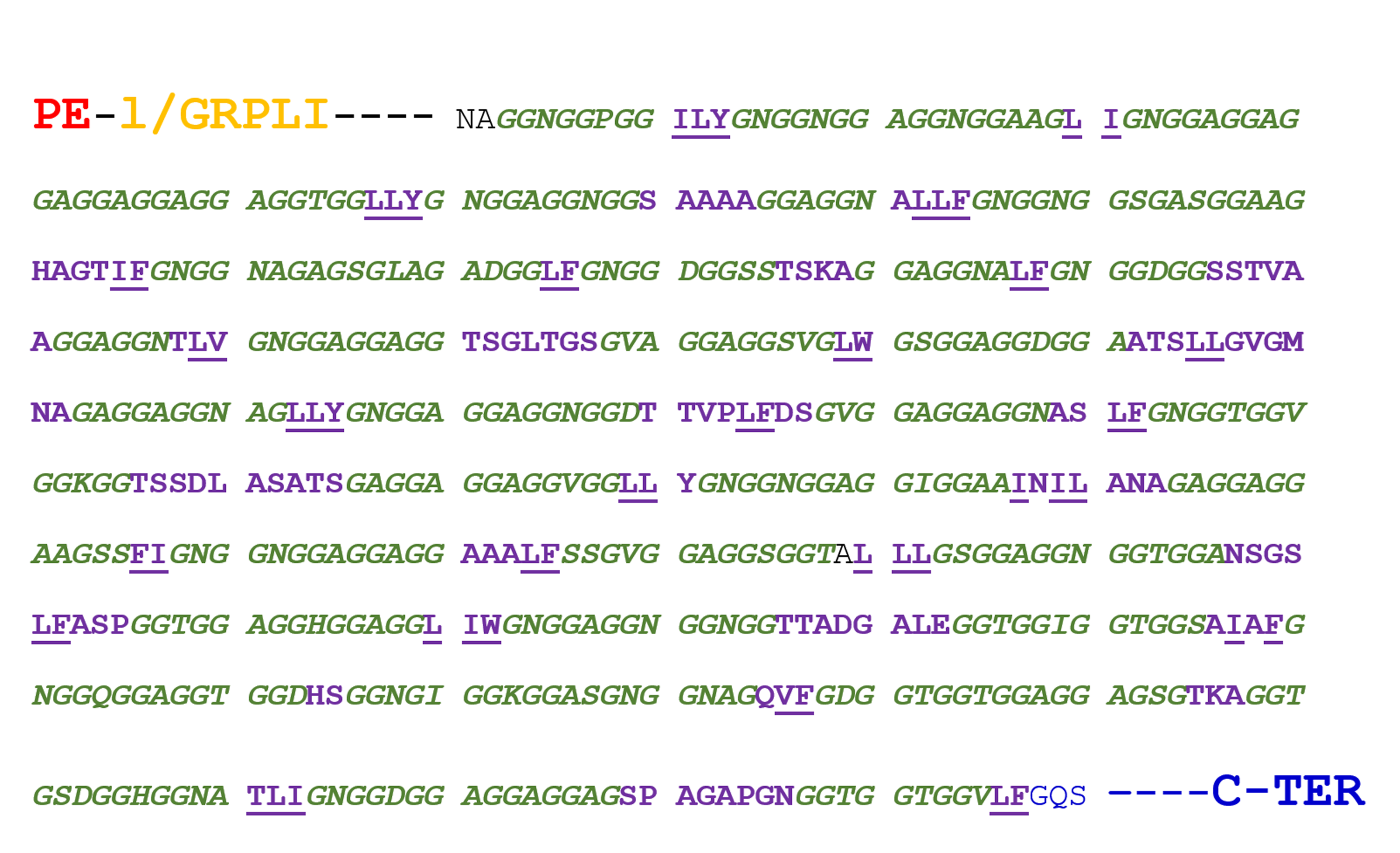

Supplement: Supplemental Material [file KVIR_A_1785815_SM4297.zip › figure S2.tif]
